# Closing the Gap: How Psychological Distance Influences Willingness to Engage in Risky COVID Behavior

**DOI:** 10.3390/bs14060449

**Published:** 2024-05-27

**Authors:** Ceridwen Williams, Paul Rauwolf, Matt Boulter, John A. Parkinson

**Affiliations:** 1 Wales Centre for Behaviour Change, Department of Psychology, Bangor University, Bangor LL57 2AS, UK; crw20zqf@bangor.ac.uk (C.W.); p.rauwolf@bangor.ac.uk (P.R.); 2 School of Health and Life Sciences, University of the West of Scotland, Glasgow G72 0LH, UK; matt.boulter@uws.ac.uk

**Keywords:** psychological distance, construal level, behavior change, COVID-19

## Abstract

Pandemics, and other risk-related contexts, require dynamic changes in behavior as situations develop. Human behavior is influenced by both explicit (cognitive) and implicit (intuitive) factors. In this study, we used psychological distance as a lens to understand what influences our decision-making with regard to risk in the context of COVID-19. This study was based on the rationale that our relational needs are more concrete to us than the risk of the virus. First, we explored the impact of social–psychological distance on participants’ risk perceptions and behavioral willingness. As hypothesized, we found that close social relationships of agents promoted willingness to engage in risky behavior. In the second phase, we tested an intervention designed to increase the concreteness of information about virus transmission as a mechanism to mitigate the bias of social influence. We found that the concreteness intervention resulted in significantly reduced willingness to engage in risky behavior. As such, communications aimed at changing the behavior of citizens during times of increased risk or danger should consider conceptually concrete messaging when communicating complex risk, and hence may provide a valuable tool in promoting health-related behavior.

## 1. Introduction

In response to the SARS-CoV-2 COVID-19 (henceforth ‘COVID’) pandemic, a core response across the world was to introduce measures to reduce human contact through social distancing [1]. For example, such measures included mandating individuals to maintain a minimum distance (often between 1 and 2 m) in public and community situations, with the aim of protecting people from exposure to viral particles contained within exhaled respiratory droplets or in the air from infected individuals [2]. Such an approach acknowledges that a critical driving force behind viral pandemics is human social interaction [3]. Humans evolved as social animals and much of our behavior revolves around the development and maintenance of relationships. Indeed, there is evidence at behavioral and neuroscientific levels that we both consciously and unconsciously follow the behavior of others [4] and derive a deep-rooted comfort and sense of security in group settings and group coherence [5,6]. More specifically, socializing is known to help maintain a sense of identity [7] and fulfilment [8]. Furthermore, social pain due to rejection has been shown to activate similar areas of the brain to physical pain [9,10] and lacking social support has been linked to an increased risk of mortality [11]. As such, understanding the key drivers of human social behavior in pandemic contexts provides an important key for a successful response and risk mitigation [3,12].

The challenge is not inconsiderable: restrictions placed on individual freedoms pose the risk of triggering psychological reactance [13,14], whereby the desire to protect personal autonomy could potentially drive people to act in ways which could be harmful to themselves, or others. This is further compounded by the political climate, including a lack of trust, contradictory messaging and a lack of observable and consistent norms for desired behaviors [15]. In a survey, conducted in the UK by Imperial College, one of many across the pandemic period, 94% of respondents said that they did not meet up with friends or family beyond their immediate household, and 95% reported that they did not have visitors to their own home [16]. However, the authors noted that these results may be vulnerable to social desirability bias, and indeed, other data suggest that adherence to government regulations was poor. When asked to report on their actual behavior over a two-week time period, only 7.2% of 681 participants reported successful compliance. Almost half of these participants (48.6%) reported intentionally breaking the rules, and this was associated with normative pressure from family and friends [17]. Furthermore, a UK study found that compliance was more closely associated with people’s practical abilities and intrinsic motivation to comply than by fear or deterrence [18]. Lower intention to socially distance has been associated with having higher self-interest, lower perceived susceptibility and being less socially responsible [17]. Taken together, these findings indicate that our decision making as it relates to minimizing the impact of COVID on our communities is heavily influenced by social and personal factors, and may be driven more by our relationship to others and our social goals than by rational evaluation of risk. Indeed, research has found that the risk of transmission appeared to be higher for interactions with friends and family than those with strangers [19] along with some evidence to suggest social norms were an important driver of social distancing behavior [20].

Social influence is argued to be strongest in situations of uncertainty, where social cues can help make decisions [21], and likewise, social norms often develop amongst an in-group to help shape appropriate behaviors for interactions and in- and out-group preferences [22]. Dual-process theory [23,24,25,26] considers social norms to have an unconscious and automatic influence over behavior that might undermine conscious and rational attempts to maintain a safe distance from others. Loewenstein and colleagues have developed a dual-process theory to account for the effects of emotion in decision-making and risk-based judgements [26]. They argue that immediate emotions, such as those triggered by the anticipation of spending time with family and friends, might bias risk judgements away from objectively more rational judgments, making decisions less sensitive to probabilities and more focused on the intensity of potential social gains. The closer the relationship, the stronger the affect, and the stronger the unconscious influence. This has been formalized in the concept of psychological distance, which offers a framework for exploring how social relationships might mediate risk behavior. Our perceived psychological distance from a person is influenced by how similar we are to them [27], how close our relationship is [28], and whether we perceive them to be part of our in-group or our out-group [27]. As such, friends and family are closer to us in psychological distance relative to strangers. Additionally, we are influenced more strongly as psychological distance reduces (i.e., proximity increases); for example, hotel guests were more likely to reuse their towels when they were told about a ‘re-use’ norm of guests at the hotel in general, but this effect increased when the norm described the prior occupants of the very room they were staying in [15,29].

In the current context, deciding whether or not to engage in an activity that would involve some degree of risk of COVID transmission requires us to balance two conflicting goals: first, our desire to sustain our relationships with others; second, to avoid virus transmission, therefore risking our own health as well as perpetuating the difficulties created by the pandemic, including restrictions on activity. In this situation, the relationship goal is likely to be more concrete, both because our friends and family are more familiar to us (psychologically proximal) and because of the emotional connection we have with them. Virus transmission, on the other hand, is likely to be more abstract, because it is something we are less familiar with and is hence more of an abstract concept.

In the first part of this study, we explored whether social psychological distance influences perceived risk and willingness to engage in behaviors considered risky for COVID transmission. Risk perception and behavioral willingness are argued to sit within the sequence of decision-making such that perceptions around outcomes and their risks comes first, and then willingness to engage in potential behaviors follows, the latter being closer in processing to actual behavior [30,31]. Additionally, we included risk perception primarily to validate our design that the differing risk levels would be perceived as such by participants. Our hypothesis was that participants will perceive the risks of interactions with friends and family members as lower compared to activities that involve strangers, and additionally be more willing to engage in behaviors with them.

## 2. Materials and Methods: Study Phase 1

**Participants. The study ran from April to May 2021, during which** 182 participants (63% female, with the majority 69% from UK, then 14% from US, and included responses from 16 different countries in total) were recruited to participate in the study, which was hosted on qualtrics.com and distributed via web link (on Instagram, LinkedIn, Facebook, and through email distribution via the authors’ and School’s networks). No specific participant targeting strategy was employed (i.e., a convenience sample). Participants who completed at least half of the scenarios were included in this analysis (*N* = 153). The study was conducted in accordance with the Declaration of Helsinki, and approved by the School of Psychology Local Ethics Committee, Bangor University (Ethics reference 16893 approved February 2021).

**Design.** This study used a within-subjects design. There were two independent variables: psychological distance (with two levels: proximal or distal) and risk level (with three levels: low risk, ambiguous risk, or high risk). In this study, we defined proximal scenarios as those that described engaging in an activity with well-known others, specifically, friends or family members. A distal scenario would be an activity involving someone or some people who are unknown, such as a taxi driver or a shop assistant. To define the three levels of risk, a conscious decision was made not to rely on current policy (e.g., the 2 meter rule), as these policies vary by country and at different time periods. Instead, we sought a more objective evaluation of risk, and leveraged the helpful gradings of risk described by Jones et al. [2] which takes into consideration multiple factors that combine to determine risk level. This allowed us to codify a range of scenarios as either low, ambiguous (medium) or high risk, and to match scenarios across levels of distance/proximity by describing the same combination of factors and only changing the agents involved.

The dependent variables were risk perception and behavioral willingness. Risk perception is defined as the subjective judgement of risk, and was measured using a 7-point Likert scale (not at all risky–very risky), in line with previous research (e.g., [32]). Behavioral willingness was chosen because it has been found to be a better predictor of future behavior than behavioral intention or behavioral expectation [31].

**Procedure.** An initial pilot study involving ten volunteers examined the variance in responses to the scenarios in order to avoid ceiling and floor effects in either the ratings for risk and willingness. From the pilot, we were able to identify 16 scenarios in total, to be divided equally across two blocks (before and after the intervention—see study phase 2). Block 1 was used for study phase 1. Participants were invited to complete the questionnaire and task online, in their own time, on their own computer or smartphone. Participants were told that this study would be examining trade-off decisions we have all had to make during the COVID pandemic, and that we were interested in understanding what people think about the relative risk of some of these scenarios. To reduce social desirability bias, we reminded participants that their responses were strictly anonymous, and that there were no right or wrong answers—each scenario representing a genuinely complex trade-off decision, and each person may have their own personal or practical reasons for the responses they choose. Participants were also told that the research would involve evaluating the risk of COVID transmission in a range of scenarios, and were asked to assume that none of the agents described in each scenario were vaccinated at this point in time. This was felt to be necessary as the vaccine roll-out in various countries across the world had begun by the time the survey was launched, and this could have influenced how people perceived their relative risk of known others vs. unknown others.

Participants reviewed the first block of eight scenarios. At the end of each scenario, the participants responded to (1) risk and (2) willingness, an example of which can be seen in Box 1.

Box 1 **Example survey item. For each of the 16 scenarios, participants were asked two questions to evaluate their risk perception and behavioral willingness.**

*Imagine the following scenario:*

 The gyms have re-opened, and you decide to go for a workout. As soon as you arrive at the gym, you run into a friend, and they suggest working out together as you haven’t seen one another in a long time.

 Assume that there are no lockdown restrictions in place, so none of the behaviors described are illegal, and to simplify it further please assume that no-one in the scenario has had the vaccine yet.

 (1) How would you describe the risk of virus transmission in this scenario? (1 = Not at all risky, 7 = Very risky)

 (2) How willing would you be to take on the risk in this scenario? (1 = Not at all willing, 7 = Very willing)

## 3. Results

**Risk perception.** To explore the effect of social distance on risk perceptions for different levels of risk, a two-way repeated measures ANOVA was conducted. We conducted analyses to understand how risk perception scores differed across different scenarios. The independent variables were risk, which had three levels (low, ambiguous, high) and proximity with two levels (proximal, distal).

A boxplot of risk perception revealed two outliers, and so these were removed before proceeding with the analysis. The number of data points included in the analysis was therefore *N* = 151. Mauchly’s test of sphericity indicated that the assumption of sphericity had been violated for both risk (χ^2^(2) = 13.92, *p* = 0.001) and for the proximity × risk interaction (χ^2^(2) = 15.10, *p* = 0.001). The Greenhouse–Geisser estimate of the departure from sphericity for risk was ε = 0.91 and for proximity × risk was also ε = 0.91. The results were therefore considered only after correcting the degrees of freedom based on the Greenhouse–Geisser correction.

The repeated measures ANOVA found a significant main effect of proximity, F(1, 141) = 9.20, *p* = 0.003, η^2^ = 0.06. On average, participants rated proximal scenarios (*M* = 4.50, *SE* = 0.08) as less risky than distal scenarios (*M* = 4.68, *SE* = 0.07). The main effect of risk was also significant (*F*(1.83, 257.61) = 571.60, *p* < 0.001, η^2^ = 0.80), indicating that participants interpreted the high risk scenarios as the most risky (*M* = 5.86, *SE* = 0.09) followed by the ambiguous scenarios (*M* = 4.85, *SE* = 0.08), followed by the low risk scenarios (*M* = 3.06, *SE* = 0.09), as we expected.

**Figure 1:**
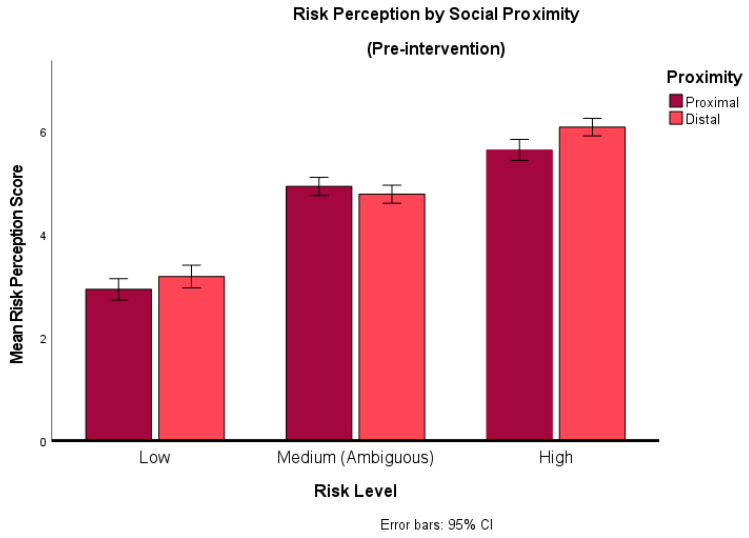
Risk perception by social distance. This graph compares risk perception for proximal vs. distal groups across scenarios that described different degrees of inherent risk (low, ambiguous, high).

The proximity x risk interaction was significant, *F*(1.8, 255.84) = 9.40, *p* < 0.001, η^2^ = 0.06. This indicated that risk perceptions differed according to the social psychological distance to the person or people described in the scenario (see Figure 1).

A paired sample *t*-test was conducted to understand this interaction further. The rating of risk for objectively low-risk scenarios was lower when they were socially proximal (M = 2.92) as opposed to socially distal (*M* = 3.18). This difference was found to be significant (t (149) = −2.21, *p* = 0.03, *d* = −0.18). Similarly, for high-risk scenarios, risk perceptions were on average lower when they were socially proximal (*M* = 5.59) compared to when they were socially distal (M = 6.01) (t (144) = −5.19, *p* < 0.001, *d* = −0.43). The reverse was found for ambiguous scenarios; when they involved socially proximal individuals, risk perceptions were marginally higher (M = 4.90) than for socially distal individuals (M = 4.73), and this difference was also significant (t (150) = 2.14, *p* = 0.03, *d* = 0.17). However, the effect sizes indicate that only the effect seen in the high-risk condition exceeded Cohen’s [33] convention for a small effect size, indicating that the differences in the other two conditions is negligible, even though they appear to be statistically significant. Therefore, we conclude that the only difference of note is for risk perceptions between proximal and distal scenarios in the high-risk condition.

**Willingness.** The differences in Willingness across low, ambiguous or high-risk scenarios involving either proximal or distal others were analyzed using a repeated measures ANOVA. A boxplot of willingness revealed no outliers. Mauchly’s test of sphericity again indicated that the assumption was violated for both risk (χ^2^(2) = 28.49, *p* < 0.001) and for the proximity x risk interaction χ^2^(2) = 13.63, *p* = 0.001), and so results were again only considered after Greenhouse–Geisser correction.

**Figure 2:**
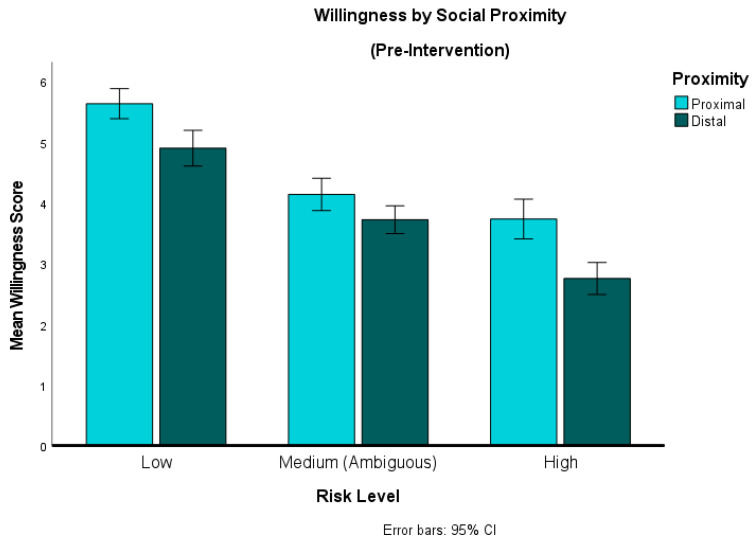
Behavioral willingness by social distance. This graph compares willingness to engage in behaviors involving socially proximal vs. distal others across scenarios (low, ambiguous or high risk).

The repeated-measures ANOVA revealed a significant main effect of proximity (F(1, 137) = 81.79, *p* < 0.001, η^2^ = 0.37) and risk (F(1.68, 230.45) = 215.73, *p* < 0.001, η^2^ = 0.61). The ANOVA also revealed a significant interaction effect of proximity × risk (F(1.83, 250.15) = 6.07, *p* = 0.004, η^2^ = 0.042) (see Figure 2).

To explore these effects further, we conducted post hoc *t*-tests, and found that mean willingness was higher for proximal than for distal scenarios across all three levels of Risk, and these differences were significant at the 0.05 level. The differences in willingness between proximal and distal scenarios was greatest in the high-risk condition (mean difference = 0.94; t (143) = 7.78, *p* < 0.001, *d* = 0.65), followed by low risk (mean difference = 0.78; t (146) = 5.92, *p* < 0.001, d = 0.49). For each of these the effect size would be considered medium according to Cohen’s [33] criteria. The difference was smallest for the ambiguous scenarios (mean difference = 0.34; t (151) = 3.21, *p* = 0.002, *d* = 0.26), a significant but small effect.

**Figure 3:**
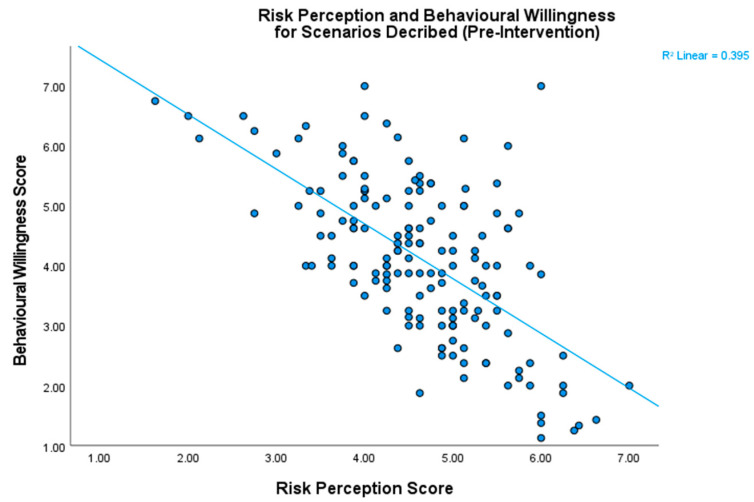
The relationship between risk perception and behavioral willingness. These two scores were negatively correlated, which validates our assumption that participants generally want to avoid engaging in situations they perceive to be risky.

Risk and willingness were found to be significantly negatively correlated (*r =* −0.63, *p* < 0.001), which served to validate our assumption (Figure 3).

## 4. Discussion of Study Phase 1 and Introduction to Study Phase 2

Participants viewed scenarios involving friends and family as less risky and showed a greater behavioral willingness to engage. The effects were greater with the behavioral willingness measure, and we observed the largest effects in the high-risk scenarios. Whilst the study successfully reflected observations in the real world, as outlined in the introduction, the study does not give a clear picture as to what is driving the effects, and hence how one might intervene to influence them. On the one hand, we have a lifetime of experience of safely interacting with our friends and family members, and familiarity is considered to be a form of close psychological proximity, whereas paucity of knowledge, for example with regard to the virus, indicates increased psychological distance [27], and processing of more abstract information has been shown to lead participants to rate events as less likely [34]. Individuals might, therefore, be making an unconscious evaluation of risk based on either past history of positive time with family, or on the likely more abstract representation of viral risk and its negative outcomes. As such, judgements might reflect the core representational nature of the events and not just their psychological distance. Psychological distance is argued to influence the ‘level of construal’, meaning the way individuals mentally represent events. Higher levels of psychological distance (e.g., future events, distant relationships) lead to more abstract and global construals, while lower levels (e.g., immediate events, close relationships) result in more concrete and detailed construals. The level of construal then acts to influence decisions in different ways. For example, concrete construals are more likely to influence immediate decisions, whereas abstract construals will have a stronger influence on future events [35]. Furthermore, if an event is perceived as being less probable we tend to view it more abstractly, and if it is perceived as expected, we view it more concretely [34].

Although our intentions tend to be aligned to our higher-order goals, we often underestimate how our behavior in the moment will be affected by low-level factors, such as how we feel [36,37]. As such, while our cognitive appraisal of the probability of an outcome tends to remain stable over time, our emotional anticipation increases as the event draws nearer and the outcome becomes more concrete [37]. When making decisions about interactions with family, the close and concrete nature of the representations may have a significant impact of our intentions. Additionally, as information about COVID is often presented in abstract terms, such as schematics of the molecular structure of a virus alongside population case numbers, we might also view it as less probable, more psychologically distant, and certainly less concrete or emotionally engaging. Therefore, by making the concept of the virus more concrete, one might increase its impact on immediate decision-making. Indeed, some recent research suggests that anthropomorphizing diseases makes them feel more proximal [38]. Therefore, we developed a brief intervention manipulating the level of construal of the virus to see if it could promote better social distancing intentions, i.e., behavioral willingness. In the study phase 2, we wanted to explore the impact of this intervention designed to increase the level of concreteness with which participants conceptualize how the virus transmits to see whether this would impact their willingness to engage in risky behaviors. As such, our hypothesis for phase 2 was that increasing the level of concreteness for transmission of the virus, through an intervention, would reduce willingness to engage in risky behavior.

## 5. Materials and Methods of Study Phase 2

**Participants.** The same participants proceeded to the second stage of the research. Participants who completed at least half of the scenarios in the second block were included in this analysis (*N* = 136).

**Design.** Initially, participants were randomly assigned to one of three groups: a control (*N* = 48), and then also two interventions groups (verbal, *N* = 41, and visual + verbal *N* = 47). The rationale behind the two intervention groups was to test two levels of ‘concreteness’ by including vivid imagery in one intervention condition, and text only in the other. However, preliminary analysis showed there was no difference between the behavior of the two groups following these interventions (all t < 2), and so, given that both forms of intervention were designed to increase concreteness, we combined these two groups into a single “intervention” group (*N* = 88), resulting in a 2 × 2 × 2 design with time having two levels (pre-intervention, post-intervention), psychological distance having two levels (proximal, distal), and another group having two levels (control group, intervention group). The dependent variable was behavioral willingness, for which part one of the study served as our baseline.

**Procedure.** Following directly on from part one, participants were now asked to complete a short task. In the verbal condition, participants were asked to read a short paragraph of text about COVID-19 transmission (see Appendix A*—Intervention*). They were then asked to answer some simple questions about the article. This served to ensure participants had engaged with the content of the intervention. In the visual + verbal condition, the same information and task was given, but this time supported by visuals. In the control condition, participants saw a neutral message (“You’re halfway through. Thank you for making it this far! When you’re ready, click to continue on to the remaining scenarios”). They then completed their second block of eight scenarios, different in content to the first set but matched in terms of the factors that determined the level of risk (e.g., masks, ventilation, number of people, and amount of time).

## 6. Results of Study Phase 2

The independent variables were time with two levels (pre- and post- intervention), proximity with two levels (proximal, distal) and group with two levels (intervention group, control group). The dependent variable was behavioral willingness. Levene’s test was not significant. A boxplot analysis revealed no outliers. As none of the independent variables exceeded three levels, sphericity could also be assumed, so we proceeded with a three-way repeated measures ANOVA.

**Figure 4:**
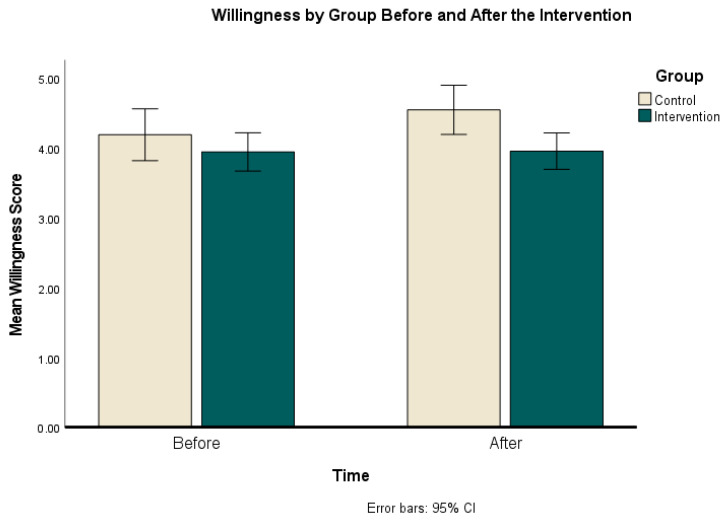
Behavioral willingness by group before the intervention (Time 1) and after (Time 2).

The results show that there was a small but significant main effect of time (F (1, 134) = 8.158, *p* = 0.005, η^2^ = 0.06). However, qualifying this effect was a significant interaction effect for time × group (F (1, 134 = 7.18, *p* = 0.008, η^2^ = 0.05); see Figure 4. 

Finally, there was also a main effect of proximity, F(1, 134) = 84.26, *p* < 0.001, η^2^ = 0.39. None of the other main effects or interactions were significant.

To explore the time x group interaction effect further, a paired sample *t*-test was used to see whether the difference in willingness from before the intervention and after the intervention was significant for each of the groups. The results showed that the control group were significantly more willing to engage in the behaviors described in the scenarios in the second block (*M* = 4.55, *SD* = 1.31) than in the first block (*M* = 4.20, *SD* = 1.39), t(47) = −3.41, *p* = .001, *d* = −0.43), an intermediate effect size. For the intervention group, the difference in willingness pre- and post- was not significant (t(87) = 0.230, *p* = 0.82).

An independent samples *t*-test was used to examine the difference between the control group and the intervention group pre- and post- intervention. The results showed that these groups were not significantly different before the intervention (t(134) = 1.091, *p* = 0.277, *d* = 0.20), but that after the intervention willingness was higher in the control group (*M* = 4.5, *SD* = 1.30) than in the intervention group (*M* = 3.96, *SD* = 1.20), t(134) = 2.67, *p* = 0.008, *d* = 0.48), with a medium effect size.

However, because there was a difference pre-intervention and a 27.7% chance that this difference was by chance, a second analysis was conducted to remove the potential confound of differences within the groups. A new variable was created for each individual’s change in willingness pre- and post-intervention, and an independent samples *t*-test was run to see whether the change in willingness was different between the two groups. The results were t(134) = 2.65, *p* = 0.009, *d* = 0.48, 95% CI [0.09, 0.59]. This indicates that the change in Willingness for each group was significantly different, and the effect size for this was small to moderate.

## 7. Discussion

Within the context of COVID and the risk to health, this study asked participants to rate how willing they were to engage in social behaviors in a variety of real-world scenarios. The key difference in the scenarios was that half were involved agents who were psychologically distant (e.g., strangers), and the other half were psychologically proximal (e.g., friends or family). While relationships to others did have a small and significant effect on perceptions of how risky a behavior was, it had a larger effect on self-reported willingness to engage in that behavior. This effect was greatest in the high-risk scenarios condition. In a second phase of the study, we manipulated construal level as part of an intervention, specifically to make representations of the COVID virus more concrete and hence psychologically proximal. Participants in the experimental group read a script (which, in some cases, included a representation; see Appendix A) that presented COVID in concrete, literal, and operational terms—as defined by construal level theory [27,34,35]. This was expected to lower the construal level of representation for information about the virus because it introduced specific details about how the virus transmits, as well as concrete examples about how wearing a mask or ventilating a space would affect this. Subsequently, participants who read the script were significantly less willing to engage in risky scenarios relative to controls, and this effect was observed across all scenarios, i.e., not restricted to either proximal or distal groups. As such, the intervention appeared to successfully influence participants such that they become less willing to engage in COVID-related risk behaviors in all scenarios.

Traditional risk-value models struggle to explain the findings from Block 1, i.e., differences in judgement for social groups, as, for example, the expected value of the goal should be the same, but decisions often systematically vary according to factors such as psychological distance [39]. Indeed, research has demonstrated how social influence can introduce an element of bias to judgements and evaluations [21]. Such biases are pervasive and form very early in our lives. For example, studies have demonstrated in-group preference in children as young as five years old [40]. A recent review [15] highlighted in-group norms as being particularly influential for adherence to COVID-19 prevention and control guidelines. In this study, it is possible that the scenarios describing proximal others conveyed information about an in-group norm and hence produced a systematic social influence towards engaging. For example, if a friend is offering you a lift in their car, the implication is that the friend does not see anything wrong with this behavior, and if you view this behavior as exemplary for the group, you could be motivated to behave in accordance with the rest of the group.

A nuanced finding was the observation that risk perception scores were lower for proximal scenarios when the level of risk was clear, but when the level of risk was ambiguous, this finding was reversed. Furthermore, differences in willingness for proximal groups were smaller for the ambiguous scenarios. Dual-process models of information processing, such as the Elaboration Likelihood Model [41], highlight the influence that our degree of engagement with information has on how it is processed. These models distinguish between central and peripheral routes of processing, and demonstrate that outcomes may differ depending on which route is activated. It is possible that, when the level of risk was obvious, participants were not required to process the information deeply; this allowed heuristics such as in-group bias to exert more influence on the subsequent ratings for risk and willingness. However, when the scenario was ambiguous, they needed to pay more attention to the detail, which may have brought the level of construal for this information down (e.g., made it more concrete), making this form of influence more powerful.

Social relationships are considered to have special psychological importance across many different aspects of psychology, and so they could be considered a form of super-ordinate goal. For example, most theories of fundamental human needs include an element of “social connection” (Maslow’s hierarchy: [42]; self-determination theory: [43]; core social motives theory: [44]), and the belief that our behavior is motivated by achieving need satisfaction or avoiding need frustration. Furthermore, we ran this study between April and May 2021, when many participants had endured months of social distancing and numerous local and nationwide lockdowns. Therefore, connectedness goals may have been particularly salient for these participants. In line with this interpretation, participants’ informal comments during the study revealed that they felt their responses were influenced by how much they valued the activity described.

Our membership to particular sub-groups such as the political party one supports has been shown to mediate our response to an energy conservation nudge [45]. In group exercise settings, where exercise is likely to be a group norm, the degree to which one identifies with the group predicts participation in the activity [46]. These are both examples of how group membership, group identity and other social or situational factors [21,47,48] may exert an influence on the efficacy of a social norm, but by what mechanism is less clear. As an example, picture an image of a crowded beach. The impact that this has on you may vary drastically depending on your interpretation of the scene. While some may decide it must be safe to go to the beach, given that many other people are going, others may conclude that the beach is no longer safe, given that so many people decided to go. It could also depend on who is on the beach, and whether they are people you know, or strangers, and whether or not you identify with the group that they belong to. The importance of how norm information is processed was highlighted in a recent study. Jiang et al. [49] investigated the impact of social norms on the relationship between news attention and social-distancing behavior, and reported that—contrary to their hypothesis—norms exerted a negative interaction effect. That is, if an individual perceived a strong norm to practice social distancing in their in-group, they may be less likely to engage in this behavior themselves, despite believing it to be effective and the consequences to be severe. On the other hand, if they did not believe that others in their inner circle were social distancing, they compensated by being more diligent themselves. While authors did not offer an interpretation as to why this effect was observed, it serves to reinforce that norms can have unexpected consequences, and also further highlights the gap in our understanding of the underlying mechanisms by which norms may influence behavior.

An explanation for our current findings based on construal level theory suggests that reducing psychological distance influences the nature of outcome evaluations. Decisions viewed as distant are typically judged by outcome desirability, whereas those seen as close are assessed by feasibility [32,35]. For example, when considering whether to attend a guest lecture, when the guest lecturer’s name was similar to their own, participants were swayed more by feasibility (how convenient it is to get there), and when the name sounded different, they were more swayed by outcome (how interesting the lecture is). By having a similar name, the psychological distance was reduced, and this shifted their preferences [50]. In this study, pre-intervention scenarios were likely considered abstractly, emphasizing outcome desirability, such as the appeal of social interactions. Post-intervention, awareness of transmission risks rendered these decisions more concrete, prioritizing feasibility over desirability and possibly reducing willingness to take risks.

Alternatively, as the concreteness intervention reduced willingness to engage across all scenarios (not just those psychologically proximal), it may have more generally been the effect of generating more intense immediate emotions that influenced all decision-making [26,37], in this case, by increasing fear of the virus and hence suppressing all behavioral intentions. Such an influence has also been described as the “immediacy bias” in that people focus on current and affectively arousing information when making immediate decisions about behavior. There are two assumptions here. The first is that the information in the intervention produced an affective response, and second, that when making the decisions, the participants were conceptualizing their responses in terms of an immediate behavior, i.e., they were making a decision about something they were going to do now, and not at some future date. It would be valuable, in future work, to explore both these assumptions. For example, whether the power of the concreteness intervention rests on increasing the affective influence on decision-making in the moment [26]. Likewise, exploring decisions for immediate behavior versus ones that will take place in the future. This would also test a further prediction of construal-level theory, which is that that whilst concrete construals impact immediate decision-making, abstract construals should have a stronger impact on future decision events [39,51]

Our study had several limitations, partially due to the rapid changes in context while it was being conducted. At the time of the study, the rollout of the COVID-19 vaccine was just beginning, meaning that we had to clarify in the instructions that participants should assume no-one in the scenarios described had been vaccinated yet. Otherwise, knowledge of friends or family’s vaccine status compared with lack of knowledge about strangers’ vaccine status could have confounded our findings. However, there is still a chance that this influenced the results. Willingness to engage in behaviors associated with the risk of virus transmission was significantly lower for the intervention group relative to the controls. Visually exploring this data, we saw that the willingness of the control group was higher for the second block of scenarios than for the first block. This may indicate that, although the scenarios were developed with a rubric to ensure they were mapped to the first block of scenarios in terms of risk variability, there may still have been some other variables that influenced the baseline level of willingness for these scenarios. This indicated that the between-group comparison may be more reliable than within groups. Importantly, there were no differences between the control group and the intervention group at baseline, meaning that the between-groups comparison was meaningful. Furthermore, we conducted a within-group analysis to control for any between-group confounds, and confirmed that willingness increased more for the control group than for the intervention group. An additional consideration with the intervention was that we originally designed two configurations, one purely written, and one with a supporting visual representation (Appendix A). The impact of the two did not differ even though we expected that the visual form might have been more impactful. The differences in presentation format warrant future exploration to determine the most impactful way to present an important message. Finally, some of our results may have been artefacts of the study design rather than a true replication of how these decisions are processed in a real world setting. For example, we opted for a simple Likert scale of not at all willing to very willing, which implies that the decision being made is binary (to engage or not to engage in the behavior). In reality, these decisions are rarely binary as there are often many different options for how to respond: for example, taking an at-home COVID test before attending a social gathering, opening a window or choosing to wear a mask, as means of adjusting the relative risk of the situation. Presenting different options at once, known as choice bracketing, has been shown to influence choice independently of construal level [32]. A final avenue for further work is that the results in the current study intervention might have reflected a general suppression of behavior (a fear response), rather than an impact specifically on COVID protective behaviors.

## 8. Conclusions

This study has shown that people are influenced in decisions about behavioral willingness by their relationships to those around them, and that concretizing representations of the COVID virus can go some way to mitigating risky behaviors. Concrete representations of the negative consequences of behavior have been widely used in attempts to reduce addictive behaviors, e.g., cigarette packaging, but the approach has been criticized as it can generate anxiety and also does not directly promote the desired behavior [52] though see [53]. However, in certain circumstances, such as during immediate decisions in uncertain circumstances, such interventions may serve to bias behavior in a safer direction [12,53]. In contrast, a natural tendency for communicating risk to groups is to use higher-level abstract construals (such as public communications) [54]. For immediate behaviors, concreteness interventions could be particularly effective in situations where there is a conflict between the desired behavior and competing super-ordinate goals such as our desire for social connectedness. There is a need here to provide a clear indication of what the ‘safe’ or desired behavior would be. Communicating risk in health messages is increasingly important for sustainability of both people and the planet. Dual-process approaches help to reveal the myriad ways human behavior is influenced, and this work demonstrates the importance of understanding behavioral drivers, in this case construal level, in designing communications. Ultimately, it serves to emphasize the importance of building capability and capacity in behavioral science in building an effective and coherent response to threats to society in an increasingly uncertain world.

## Figures and Tables

**Figure A1:**
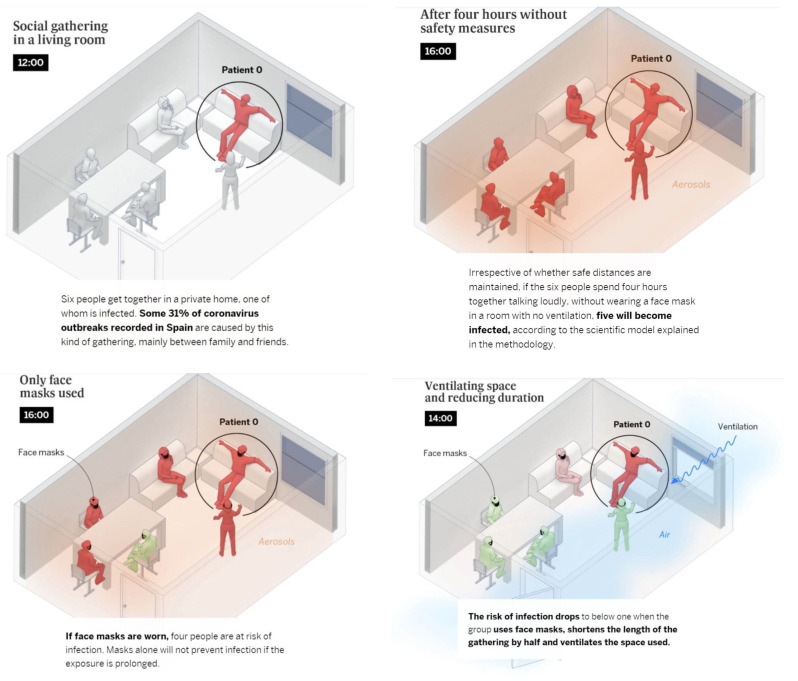
Intervention Materials.

## Data Availability

Where relevant, all anonymized primary data is available via the corresponding author.
